# Combining Persuasive Technology With Behavioral Theory to Support Weight Maintenance Through a Mobile Phone App: Protocol for the MotiMate App

**DOI:** 10.2196/resprot.4664

**Published:** 2016-01-08

**Authors:** Emily Brindal, Gilly A Hendrie, Jill Freyne

**Affiliations:** ^1^ CSIRO Food and Nutrition Adelaide Australia; ^2^ CSIRO Health and Biosecurity Marsfield Australia

**Keywords:** app, weight maintenance, psychology, coping

## Abstract

**Background:**

The prevalence of health-focused mobile phone apps available for download increases daily, with weight management apps being among the most proliferative. However, most lack theoretic grounding or evidence of efficacy. There is a significant body of literature which provides evidence for behaviors which are associated with successful weight loss maintenance. Behavioral theory also provides further insight regarding successful behavior change and maintenance.

**Objective:**

We aimed to apply this knowledge to the development of the functionality of an app targeting weight loss maintenance.

**Methods:**

We have subsequently undertaken the development of a persuasive and behavior targeting mobile app (MotiMate) to assist in maintenance of weight loss. MotiMate combines persuasive and behavior change theories in a practical targeted tool through its motivational messages, personalized feedback, and intelligent supportive tools to manage weight, food, exercise, mood and stress.

**Results:**

The development and trial of MotiMate received funding support in May 2014. All 88 volunteers started the trial by December 2014 and were in the process of completing their final visits when this paper was submitted (May 2015). Data analysis is currently underway.

**Conclusions:**

The paper has presented a scientifically informed mobile phone app to support weight loss maintenance. Further evaluation of its efficacy is in progress.

**Trial Registration:**

ANZCTR 12614000474651; https://www.anzctr.org.au/Trial/Registration/TrialReview.aspx?id=366120 (Archived by WebCite at http://www.webcitation.org/6eJeQiKxi).

## Introduction

Thirty percent of the global population is overweight or obese, with this predicted to rise to almost 50% by 2030 [1]. Many people have success in changing their dietary and/or physical activity behaviors to lose weight; however, few successfully maintain their lost weight over the longer term. In order to gain the multiple health benefits of weight loss (such as improved heart health), lost weight must be maintained. A review of The National Weight Control Registry in the US found only 20% of people managed to maintain initial losses after 2 years. Those members reporting maintained weight loss engaged in more physical activity, monitored their weight regularly and maintained a consistent eating pattern throughout the week [2]. If individuals can successfully maintain their weight loss for 2-5 years then the chance of longer-term success increases greatly [3].

To promote the behavior change needed for weight control, an interactive device such as a mobile phone has the capacity to intervene at critical times or provide ecological momentary intervention (EMI). A review of EMIs using portable devices identified 5 papers that have targeted weight loss [4]. All of these systems were designed to offer behavior change techniques (such as goal setting and monitoring) and keep users motivated, with some focus on the provision of feedback and others giving psycho-education. The review authors concluded that EMI through portable devices may be useful to enhance cognitive-behavioral therapy for weight loss programs. There was limited evidence evaluating the effectiveness of EMI delivered in the absence of other more intensive programs. A separate review focusing specifically on mobile phones suggested that existing systems have underutilized the functionality of modern phones, by relying on short message service (SMS) and not providing immediate two-way feedback, for example [5].

Despite a high number of weight control apps publicly available, few are scientifically derived [6]. Furthermore, despite the fact that theoretically based interventions may be more efficacious for behavior change [7], authors reviewing mobile phone interventions conclude, “a paucity of discussion regarding the health behavior theories or models that provide the basis of intervention” [7].

Behavioral theory can offer several insights and more in-depth understanding of how to achieve successful behavior change when in combination with apps. Conservation of resources and self-regulation theory suggest that an individual has limited capacity to navigate stresses successfully through each day and to do that, people must utilize the appropriate resources [8,9]. These extend beyond an individual’s capacity to control their own behavior to other more tangible resources such as social support and even practical things such as money [8]. The more resources one has in their possession or reach, the more likely that they will be able to use them to avoid stress, overcome barriers, self-regulate and achieve desired behaviors. For example, a person trying to limit intake of fatty snacks may run out of psychological resources to resist temptation (self-regulation) but could then use other resources such as friends to keep motivated (social support). There is evidence that people low in psychological resources to deal with hassles may be more vulnerable to illness [10] which suggests that resources are important for well-being. There are clear intersections between the concept of limited resources and the potential benefits of adaptive coping strategies. For example, coping strategies could become a resource for an individual to use to overcome their challenges [11]. Considering that people may try 6-7 strategies when addressing a hassle [12], it is not surprising that the more coping strategies or resources a person possesses, the more likely it is that they will find one that successfully helps them to sustain their desired behavior change.

The ability to recognize, address and cope with the multiple challenges that come with behavior change is an essential part of long-term success. For weight maintenance, the possible coping scenarios for individuals in different situations are almost infinite. Removing focus from behavioral minutiae toward one’s global capacity to ‘cope’ with emotional or stress changes throughout the day may be a more practical way to assist people with behavioral maintenance and weight control. This can be done in three ways, the first of which is equipping the individual with a wider set of strategies. There is evidence that the more resources a person possesses, the more resources they are likely to accrue [13]. A second strategy would be through improving a person’s psychological well-being. Positive well-being and optimism can improve resilience and the ability to problem-solve [14,15], as well as helping to restore resources after depletion [16]. Finally, a greater feeling of control over coping could equip a person with more confidence to use strategies. The Health Action Process Approach (HAPA) [17] suggests that self-efficacy (confidence) to initiate and maintain behaviors is critical to behavior change as people transition from action planning to coping planning. In other words, once the plan to lose weight has been initiated, individuals need to move on toward more coping-oriented planning in order to maintain healthy behaviors.

Thus, a supportive mobile phone app can be used to provide basic behavioral therapy with the intention of equipping people with greater self-awareness and better coping strategies, which, according to theory, could be associated with long-term behavior change. In some recent studies, a weight loss maintenance intervention using intelligent technology to assist people with diabetes to monitor their weight reported that higher use was associated with better weight loss results [18]. In a pilot trial, a behaviorally based iPhone app significantly improved psychological outcomes (mood and motivation) on a 2-month weight loss program [19]. However, no existing program grounded in behavioral theory has incorporated simple weight loss maintenance strategies into a supportive program that also targets well-being.

This paper will describe the development of a mobile phone app designed to use behavioral theory to target weight maintenance. By describing each of the core components and how they are intended to function, we aim to provide empirical and theoretical rationales for the inclusion of each of the app components as well as guidance for other developers regarding a potential approach for the development of app-based behavior change interventions.

## Methods

### Key Features of a Behaviorally Based Mobile Phone App for Weight Maintenance

We have attempted to design an app that aims to improve a user’s personal coping resources through basic behavioral therapy techniques that encourage workshopping of resources to deal with different moods and stresses while also providing weight, diet and exercise monitoring and utilizing immediate two-way feedback. Key features of the MotiMate app along with the description of how they fit with theory and/or existing scientific evidence are described briefly below.

#### Behavioral Prompts

Simple SMS prompts can be useful for short-term behavior change [20]. There is also strong evidence to support the benefits of prompting and automated reminders to improve engagement with an intervention [21]. MotiMate prompts participants through push notification messages to enter data daily and review feedback weekly.

#### Monitoring and Reviewing

Self-monitoring is a key behavior change technique associated with successful behavior change [22] and weight control [23]. Setting and reviewing goals are core features of most behavioral treatments which would not be possible without a level of behavioral monitoring.

**Figure 1:**
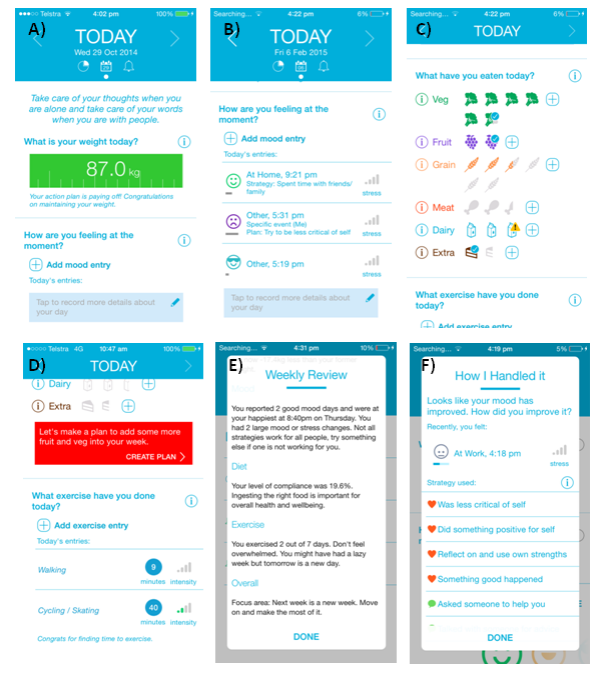
Screenshots of the MotiMate smartphone app. (A) opening page including motivational statement and weight entry; (B) Mood entries with description of hassles and coping strategies; (C) Food entries with examples of meeting and exceeding targets; (D) Exercise entry summary with prompt for diet action plan; (E) Weekly review (behavioural review); (F) Example of coping workshopping for improved mood.

Monitoring tools were designed to encourage low intensity (<10 seconds) and frequent interactions, as can be seen in Figure 1A-D, are at the forefront of the user experience as ‘homepage’ of the app.

#### Weight Monitoring

Multiple studies have reported on the benefits of weight monitoring for weight control [24,25]. Real-time two-way feedback is displayed below the weight data as it is entered. This feedback is tailored based on existing definitions of weight maintenance: maintaining, danger zone and gaining [26]. Classification into one of these categories determines the nature of the textual feedback that people are given as well as the color of the weight display which, as can be seen in Figure 1A, progresses from green (maintaining) to dark green (danger zone) to grey (gaining). As participants enter the danger zone, the short automated message contains content designed to motivate them to stay on track. If they enter a gaining zone, the tone of these messages changes, remaining encouraging but becoming more directive, and an email is also sent to inform the administrator of the possible need for further intervention. Examples of messages for maintaining, danger zone and gaining messages respectively are: “Well done! You have maintained your starting weight. Keep it up,” “You are a little heavier than your starting weight. Just a bit more effort this week and you will be back on track,” and “You've gained some weight, try reviewing your lifestyle habits and keep keeping track of your weight.”

#### Well-Being

Well-being monitoring was included to allow people to track their moods, so that they can see any link between emotional states and other behaviors (in summary sections) but also to allow a point of EMI for the coping behavioral support tool. Previous apps have tried to capture this style of processes successfully without persuasive features [27]. Mood states included were designed to capture pleasant and unpleasant moods as well as moods with high versus low arousal [28]. A user also identifies emotional intensity from 1-10 and location and time to allow review of data and identification of potential patterns in mood and to assist in the classification of coping thoughts and actions [29]. Stress (captured independently to mood) is measured through an intensity bar. No feedback text is presented below mood entries. The system detects and alerts an administrator via email if participants appear at psychological risk. The administrator’s email address is unique to the app and monitored by the project manager for the trial version of the app. This person directs alerts to the appropriately qualified staff within the research institution (nutrition for weight gain and psychology for mood changes). Psychological risk is calculated by summing the intensity scores for each mood entered each day (with pleasant and unpleasant moods associated with positive and negative scores respectively). A summed negative score is used to indicate an “unhappy” mood day. The system sends an alert for a severely unhappy day (a summed score 3 standard deviations from the mean) or prolonged “unhappy” days (7 consecutive days).

#### Diet and Exercise Monitoring

Given consistent evidence about the link between diet, exercise and weight loss maintenance [2], the app includes tools to monitor these behaviors. The aim is to design an approach that is generic enough to support a variety of different approaches for diet and exercise regimes that individuals had adopted in their weight loss journey. Therefore, national recommendations were used to provide feedback and set targets [30,31]. On creating an account, users are provided with personalized daily targets for each food group (e.g., fruit, vegetables, meat and dairy foods), displayed as grayed-out icons. If intake exceeds the target for a day, then an exclamation symbol appears for all food groups except fruit and vegetables. As can be seen in Figure 1C, to positively reinforce good eating habits, a tick appears if the user meets their target for all food groups (except for the junk food category, as less is always better). Figure 1D shows how automatic textual feedback is given on exercise records based on a series of rules formed using the Australian National Physical Activity Guidelines for adults and how many entries the user had made for the day.

In order to help keep users on track with their diet, an action planning interface is embedded in the food intake monitoring interface. When food intake consistently exceeds or falls below recommendations, users are provided with a predefined list of actions to choose from to help them “get back on track” and meet their food group targets.

#### Reviewing Data

Data from monitoring tools are collated into summary graphs to allow the users to reflect on their behaviors and identify potential patterns, as illustrated in Figure 1E. Once a week a behavioral review is also released that summarizes the user’s weekly performance and gives feedback on which category a user is doing well on as well as their ”focus” area (i.e. an area where they are performing poorly). Constructive reviews are critical in many behavioral therapies.

#### Behavioral Support and Intervention

Supportive features in the app include motivating messages, personalized feedback in monitoring tools (as described above), and the coping tool. Previous studies have reported that supportive features can be beneficial for mood amongst women losing weight [19]. While some factors (eg, goal setting, motivation, and self-efficacy) may promote adherence to weight loss programs, psychological factors, such as stress and depression may inhibit peoples’ ability to maintain weight losses [32-34]. Daily motivational messages were constructed based on inspirational quotes and thoughts to inspire optimism and the potential for higher resilience and problem-solving ability [14]. In accordance with HAPA theory, and as Figure 1A shows, these messages transition from a focus on general motivation and action planning through to coping planning.

#### Coping Behavioral Therapy Tool

Psycho-education contained within the information buttons provides guidance on maintaining positive mental well-being based on positive psychology and cognitive therapy. This component is designed to promote self-awareness and equip people with greater self-efficacy to recognize and modify their behaviors in order to maintain their positive behavior changes.

The coping workshopping interface only appears once a large change in mood or stress is detected from the data entered. In order to capture information about both potential triggers and coping resources, the interface appears for both negative changes in mood or stress (eg, a decrease in happiness of 2 standard deviations or greater based on the group mean; or a good to a bad mood) and when mood or stress levels improve (eg, increase in happiness or a bad mood to good mood). Focusing on the coping resources that users have successfully applied rather than simply how they could fix hassles is an important behavioral element in the effort to assist users in building effective coping strategies.

Once the hassle interface is triggered, participants enter data on the nature of the issue, to whom it relates, and select a coping strategy they could use to address the issue. A range of coping strategies are presented in a predefined order using an algorithm assessing user appraisals of the hassle, including the immediacy of the issue, a user’s perceived feeling of control and whether or not they want to fix the issue. Strategies are grouped generally into categories: social support (eg, “Talk with someone for advice”), personal strengths (eg, “Reflect on and use own strengths”), distraction (eg, “Avoid the issue”) and emotional or cognitive strategies (eg, “Change my thinking about it”) versus active strategies (eg, “Make a plan”). Escape/avoidant strategies could be associated with negative psychological symptoms [35] and therefore always appear at the bottom of this menu. When a positive change in stress or mood is entered, as Figure 1F shows, participants are asked what strategy they used to change their mood. In all menus, the option to add free-text to indicate something else is available. Once a person has workshopped their coping strategies, Figure 1B shows how the data are summarized on their home screen once a person has workshopped their coping strategies. Terms such as strategies and ‘how I handled it’ are presented to users in an effort to avoid negative pre-conceptions related to ‘coping’ [36].

## Results

The development and trial of MotiMate received funding support in May 2014. All 88 volunteers started the trial by December 2014 and were in the process of completing their final visits when this paper was submitted (May 2015). Data analysis is currently underway and the first results are expected to be published early in 2016.

## Discussion

### Principal Findings

The paper has presented a scientifically informed mobile phone app to support weight loss maintenance. Many papers exist that propose or discuss behavioral theory and how this could be used to develop behavior change systems. Putting this into practice is challenging as it is often difficult to incorporate the number of features and depth required in such an evidence-based approach without compromising user interaction. We have used theory to guide the focus and ethos of MotiMate while also incorporating core behavioral therapy techniques, such as monitoring and reviewing. This has required drawing on insights from multiple theories (i.e., Conservation of Resources Theory) and models (i.e., HAPA) as well as scientific evidence from the literature. Despite this depth, we have attempted to keep user interactions simple by including multiple persuasive features. In an effort to facilitate shorter, more frequent interactions, the app does not collect detailed data. This means reliance on predetermined options and categories and a minimum level of user literacy. At this stage we have used the literature to guide the development of categories and provided “other” options where possible, but as more user data is collected, these categories will become more intuitive. More icons and graphical feedback may need to be incorporated into future versions to reduce reliance on high literacy levels for the two-way feedback.

### Future Work

The MotiMate app is currently being trialed as part of a Randomized Control Trial to assess its efficacy (ANZCTRN 12614000474651). The trial requires participants to visit a clinic on 5 occasions over 24 weeks to have their weights taken and complete several psychological questionnaires. Data from this trial will help us to assess its efficacy as a persuasive tool for maintaining positive psychological well-being and weight relative to less supportive app. Detailed user feedback for each of the core features will be captured at weeks 4 and 12 to allow us to understand the relative strengths and weaknesses of each of the features. Usage logs of app features and correlation to behavior change are also being recorded throughout the trial. This will allow us to understand engagement over time relative to a basic monitoring app. It will also facilitate evaluation of how app use may change relative to changes in psychological well-being (such as increases in stress or depression) that will be collected during visits to the clinic. In a real-world application (outside the research institution), the alert system will require an administrator who can refer users to local primary care health services. The potential ongoing workload associated with this will also be evaluated to ensure its feasibility. In summary, the controlled trial will be a powerful method to allow assessment of the efficacy of the app for behavior change, capture app usage patterns, receive detailed user evaluation feedback, and assess feasibility of commercially releasing the app.

## Abbreviations

EMI: ecological momentary intervention
HAPA: Health Action Process Approach
SMS: short message service
